# The complete chloroplast genomes of two *Pedicularis* species (Orobanchaceae) from Southwest China

**DOI:** 10.1080/23802359.2022.2080018

**Published:** 2022-06-10

**Authors:** Wei-Jia Wang, Rong Liu, You Wu, Hong Wang, Wen-Bin Yu

**Affiliations:** a Key Laboratory for Plant Diversity and Biogeography of East Asia, Kunming Institute of Botany, Chinese Academy of Sciences, Kunming, China; b University of Chinese Academy of Sciences, Beijing, China; c Center for Integrative Conservation, Xishuangbanna Tropical Botanical Garden, Chinese Academy of Sciences, Menglun, China; d Center of Conservation Biology, Core Botanical Gardens, Chinese Academy of Sciences, Menglun, China; e Southeast Asia Biodiversity Research Institute, Chinese Academy of Science, Nay Pyi Taw, Myanmar

**Keywords:** Chloroplast genome, Orobanchaceae, *Pedicularis*, plastome, *Pedicularis cephalantha*, *Pedicularis nigra*

## Abstract

We report the complete chloroplast genome (plastome) sequences of *Pedicularis cephalantha* (147,087 bp) and *P. nigra* (145,726 bp), endemic to southwestern China. Both plastomes have typical quadripartite structures with one large-single copy region, one small-single copy region, and two inverted repeat regions. Both plastome sequences contained 37 tRNA genes and eight rRNA genes, but they differed in the numbers of protein-coding genes: *P. cephalantha* had 76 functional genes and 12 pseudogenes while *P. nigra* had 74 functional genes and 13 pseudogenes. Phylogenetic analysis shows that *P. cephalantha* and *P. nigra* are closely related, then sister to *P. oederi* in the family Orobanchaceae.

*Pedicularis* L. (Orobanchaceae) is the largest genus of hemiparasitic plants (c. 500 species; Yu et al. 2015) and shows remarkable diversification in corolla architecture and floral presentation. Approximately, 360 species are native to China with two-thirds endemic to the Himalaya-Hengduan Mountains region (Yang et al. 1998; Yu et al. 2015). Most previous molecular phylogenies of *Pedicularis* were based primarily on short DNA markers (Ree 2005; Yang and Wang 2007; Robart et al. 2015; Yu et al. 2015). However, chloroplast genome sequences provide additional information for clarifying molecular genetics, species delimitation, and phylogenetic reconstruction, so *Pedicularis* plastomes have been sequenced and used to build phylogenies of this genus (Li et al. 2021). In this study, we completed whole genome sequencing and *de novo* assembly of the chloroplast genomes of *Pedicularis cephalantha* Franch. ex Maxim. 1888 and *Pedicularis nigra* (Bonati) Vaniot ex Bonati 1921. We then reconstructed a plastome phylogeny within the genus by combining available plastome data of *Pedicularis* species in GenBank to determine the phylogenetic positions of both species. Voucher specimens of *P. cephalantha* (Liu R. et al., LR202070703) and *P. nigra* (Yu W.-B. et al., LIDZ1331) were collected from the Yulong Mountain, Lijiang, Yunnan, China (108°13′E, 27°00′N) and from Gaolushan, Yuxi, Yunnan, China (102°36′E, 24°50′N), respectively. Voucher specimens of the two species were deposited at the Herbarium of the Kunming Institute of Botany, Chinese Academy of Sciences (KUN, http://www.kun.ac.cn, Prof. Zhu-Liang Yang, fungi@mail.kib.ac.cn, Yunnan, China).

Total genomic DNA was extracted from leaves of both species dried with silica gel using a modified CTAB method (Doyle and Doyle 1987). For library construction using the Kit protocol (NEBNext Ultra II DNA Library Prep Kit for Illumina), purified DNA was fragmented to 300–500 bp (Zeng et al. 2018). Illumina Hi-Seq 2500 was used to construct the library with 150 by pair-end reads. Around 21,775,108 reads (12,293,748 for *P. cephalantha* and 9,481,360 for *P. nigra*) were obtained. These raw data were used to assemble the complete plastome sequences *de novo* using the GetOrganelle toolkit (Jin et al. 2020). Read mappings showed coverage depths of ∼455× in *P. cephalantha* and 805× in *P. nigra*. The chloroplast genomes were annotated using Geseq with the MPI-MP Reference Set (Tillich et al. 2017), then manually adjusted the start and stop codons of protein-coding genes using Geneious (Kearse et al. 2012). The newly assembled plastomes from *P. cephalantha* (NCBI accession: OL606628) and *P. nigra* (NCBI accession: OL544940) showed typical quadripartite structures with one large-single copy region, and one small-single copy region separated by two inverted repeat regions. Plastome sizes of *P. cephalantha* and *P. nigra* were 147,087 bp (GC content: 38.4%; LSC: 82,451 bp; SSC: 14,394 bp; IR: 25,121 bp) and 14,726 bp (GC content: 38.4%; LSC: 82,006 bp; SSC: 15,116 bp; IR: 24,302 bp), respectively. The total gene contents were 133 in *P. cephalantha* and 132 in *P. nigra*. Both plastomes had 37 tRNA genes and eight rRNA genes, but the numbers of protein-coding genes differed: *P. cephalantha* had 76 functional genes and 12 pseudogenes while *P. nigra* had 74 functional genes and 13 pseudogenes.

To clarify the phylogenetic positions of these two species, we downloaded an additional 29 published plastomes of 22 *Pedicularis* species from GenBank. These complete plastome sequences with one IR region were aligned using MAFFT (Katoh and Standley 2013). The plastome of *Phtheirospermum japonicum* (Thunb.) Kanitz (Orobanchaceae) was selected as the outgroup. A maximum-likelihood tree was reconstructed using raxmlGUI (Edler et al. 2021) with GTRGAMMAI model by running 1000 bootstrap replicates. Our phylogenetic analyses showed that *P. cephalantha* and *P. nigra* formed a clade with 100% bootstrap values (Figure 1), representing Clade 3 as in the previous study (Yu et al. 2015). Our new clade is sister to *P. oederi* Vahl in the Clade 7. The complete chloroplast genomes and phylogenetic results in this study provide new insights into the phylogenetic backbone and evolutionary biology of *Pedicularis*.

**Figure 1.**
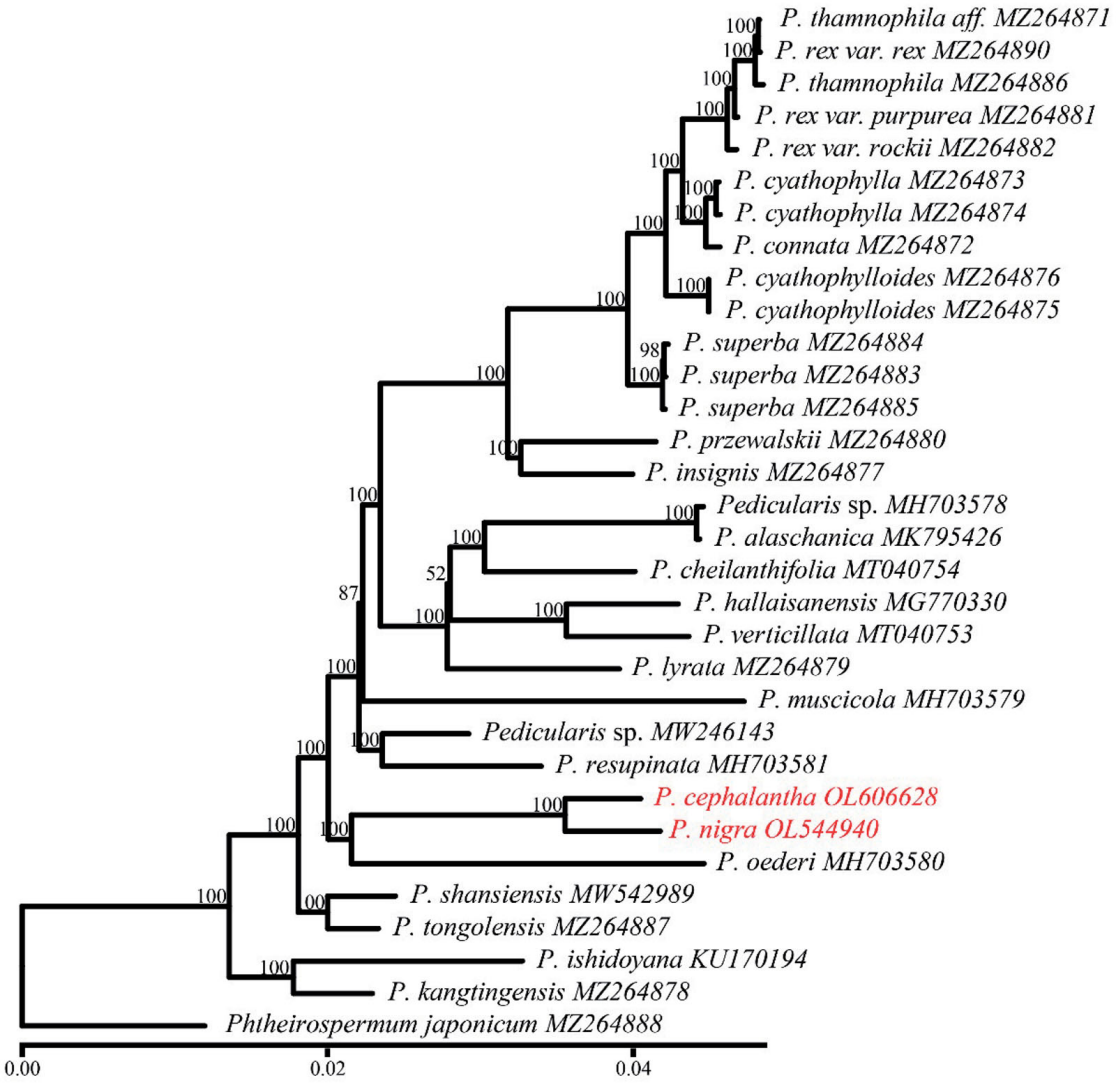
Phylogeny of 24 *Pedicularis* species including three varieties of *P. rex* based on chloroplast genomes using maximum-likelihood methods with bootstrap values on the branch. The bottom scale bar represents the number of substitutions per site.

## Data Availability

The data were collected without violation of the protection of human subjects, or other valid ethical, privacy, or security concerns. Two complete plastome sequences were deposited in GenBank with accession numbers OL606628 and OL544940 and are also available at Figshare (https://doi.org/10.6084/m9.figshare.17097098.v2). The associated BioProject, SRA, and Bio-Sample numbers are PRJNA780958, SRR16960915–SRR16962222, and SAMN23224351–SAMN23224352, respectively.
